# Rupture utérine sur utérus sain: complication du misoprostol (à propos d’un cas et revue de la littérature)

**DOI:** 10.11604/pamj.2018.31.223.12906

**Published:** 2018-12-06

**Authors:** Meryem Belmajdoub, Fatima Zohra Fdili Alaoui, Hikmat Chaara, Abdelilah Melhouf

**Affiliations:** 1 Centre Hospitalier Hassan II, Service de Gynécologie et Obstétrique 2, Fès, Maroc

**Keywords:** Rupture utérine, début 3ème trimestre, utérus non cicatriciel, misoprostol, Uterine rupture, beginning of the 3rd trimester, unscarred uterus, misoprostol

## Abstract

La rupture utérine sur utérus sain au 2^ème^ et 3^ème^ trimestre de grossesse est une complication obstétricale rare et grave mettant en jeu les pronostics vitaux maternels et fœtaux et le devenir obstétrical des patientes en l'absence de diagnostic et de prise en charge immédiate. Elle survient majoritairement sur un utérus cicatriciel et reste anecdotique sur un utérus sain. Nous rapportons le cas d'une patiente primigeste ayant présenté une rupture utérine sur un utérus non cicatriciel lors d'un déclenchement de travail par le misoprostol pour mort fœtale in utero (MFIU) sur retard de croissance intra utérin (RCIU) et oligoamnios sévère à 31 semaines d'aménorrhée (SA). Nous discutons à travers ce cas et la revue de la littérature, l'extrême prudence qu'il faut garder pour utiliser le misoprostol en cas de déclenchement du travail ainsi que les signes d'appel cliniques, les facteurs de risque, la méthodologie diagnostique et la prise en charge thérapeutique de cette entité rare mais potentiellement grave.

## Abstract (en)

Uterine rupture in patients with healthy uterus during the 2^nd^ and 3^rd^ trimester of pregnancy is a rare and severe obstetric complication involving maternal and fetal vital prognosis as well as obstetrical outcome of patients in the absence of diagnosis and early treatment. It mainly occurs in women who have had scarred uterus and it is anecdotal in those with healthy uterus. We report the case of a primiparous patient with unscarred uterus who had had uterine rupture during labour induction with misoprostol for fetal death in utero (FDIU) due to intrauterine growth retardation (IGR) and severe oligohydramnios at 31 weeks of amenorrhea (WA). This study and the literature review highlights the importance of administrating misoprostol for labour induction with extreme caution as well as the clinical features, the risk factors, the diagnostic method and the therapeutic treatment of this rare but potentially serious condition.

## Introduction

La survenue d'une rupture utérine lors du déclenchement du travail est un accident rare (moins de 1% des cas) mais potentiellement grave, pouvant conduire à l'hystérectomie d'hémostase. Le misoprostol pour le déclenchement du travail dans les interruptions médicales de grossesse (IMG) du 2^ème^ et 3^ème^ trimestre est utilisé en routine par la plupart des équipes françaises malgré l'absence d'Autorisation de Mise sur le Marché (AMM) dans cette indication. Nous rapportons le cas d'une patiente ayant présenté une rupture utérine sur utérus sain lors du déclenchement du travail par misoprostol, en l'absence des facteurs de risques habituels pouvant faire redouter la survenue d'un tel accident.

## Patient et observation

Madame FS, 29 ans, primigeste sans antécédent particulier, référée dans notre formation pour prise en charge d'un oligoamnios sévère avec RCIU sur grossesse de 30SA+2jours. Examen clinique à l'admission a objectivé une patiente consciente stable sur le plan hémodynamique et respiratoire avec une tension artérielle à 11/6 labstix positive à 4 croix, examen gynéco-obstétrical: une hauteur utérine petite par rapport à l'âge gestationnel avec des bruits cardiaux fœtaux présents et réguliers chez une patiente en dehors du travail. Un bilan de pré éclampsie a été fait qui est revenu normal sauf la protéinurie urinaire de 24h (PU24H) qui était franchement positive à 4,5g/24h avec une fonction rénale correcte, échographie obstétricale: RCIU sévère avec anamnios et estimation du poids fœtal à 524g. La patiente a été mise sous surveillance clinique biologique éclectique et radiologique, l'évolution a été marquée par l'aggravation de la PU24H: 10/4,5g avec une tension artérielle qui était toujours correcte, diurèse conservée et un bilan de pré éclampsie de contrôle qui était correcte, le Rythme Cardiaque Fœtal (RCF) était micro oscillant aréactif. Le diagnostic d'un RCIU sévère avec anamnios sur néphropathie maternelle sur grossesse de 30SA a été retenu, 3 jours après, l'évolution a été marquée par la survenue d'une MFIU. Selon les recommandations de FIGO 2012, le déclenchement par misoprostol a été effectué à la dose de 25μg par voie vaginale chaque 6H pendant 24h (4poses = 100μg), 6h après la 4^ème^ prise la patiente a commencé à avoir des contractions utérines mais qui sont espacées sans modifications cervicales ni rupture de la poche des eaux, le lendemain matin soit à 24h de la 4^ème^ prise, les contractions utérines sont devenues plus rapprochées et plus intenses avec rupture de la poche des eaux, 2 heures après la patiente a accusé des douleurs pelviennes continues avec au toucher vaginal un col dilaté à un doigt effacé à 30% avec la poche des eaux rompue et présence d'un saignement minime de faible abondance. L'examen ne montrait, par ailleurs, pas d'altération de l'état hémodynamique.

**Figure 1:**
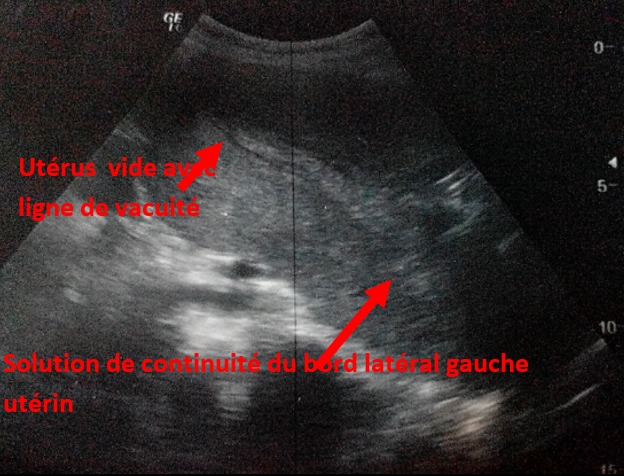
aspect échographique: utérus vide avec un hématome latéro-utérin gauche avec solution de la continuité utérine en regard

**Figure 2:**
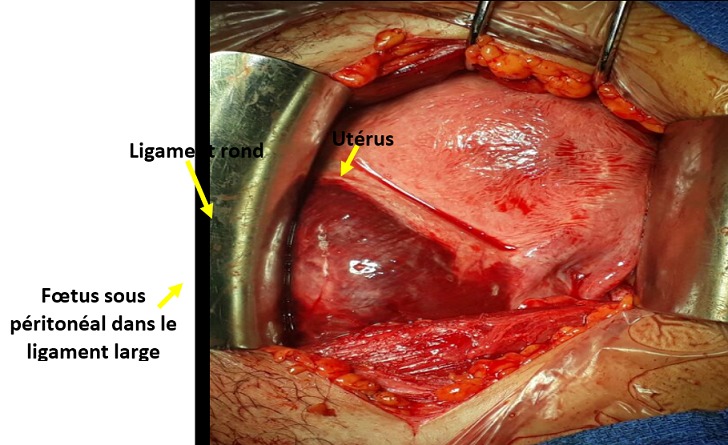
image per opératoire d’un fœtus sous-péritonéal situé dans le ligament large gauche

Une rupture utérine a été suspectée vu le saignement et une échographie pelvienne a été immédiatement réalisée ayant objectivé un utérus latéro-dévié à droite, présentant une solution de continuité du bord gauche, et un fœtus extra utérin sans activité cardiaque (Figure 1). Une laparotomie a été réalisée en urgence, permettant de confirmer le diagnostic de rupture utérine. Avec à l'exploration pas d'hémopéritoine avec un fœtus sous-péritonéal situé dans le ligament large gauche (Figure 2). La rupture s'étendait de l'isthme à la partie antérieure de l'insertion du ligament rond. Pédicule utérin gauche étant intact. Après extraction du fœtus et du placenta, nous avons décidé un traitement conservateur de l'utérus vu que la patiente est primigeste. Une ligature du pédicule utérin gauche a été faite avec suture de la rupture utérine par du fil résorbable. Les suites opératoires ont été simples et la patiente a pu quitter le service au septième jour postopératoire sous contraception orale avec un traitement martial et lettre pour consultation de néphrologie pour une ponction biopsie du rein.

## Discussion

La rupture utérine est une solution de continuité complète du mur utérin ainsi que de sa séreuse. La lumière utérine communique alors avec la cavité péritonéale. On distingue deux types de rupture utérine (RU): traumatique et spontanée. Les étiologies de RU dites « traumatiques » sont variées et peuvent être en rapport avec un choc (direct ou indirect) ou des manœuvres obstétricales (manœuvres endo-utérines ou expression utérine). Nous nous intéresserons plus particulièrement aux RU dites spontanées qui surviennent en dehors de tout contexte traumatique [1, 2]. La rupture utérine est considérée comme un accident rare dans les pays développés, survenant dans 1/2.000 naissances, alors que son incidence est beaucoup plus élevée dans les pays en voie de développement, atteignant 1/100 naissances [3], ce qui reflète la différence des conditions socio-économiques et des niveaux de surveillance médicale. En effet, c'est l'insuffisance de personnel qualifié et d'infrastructures sanitaires qui serait à l'origine de cette différence [2, 4]. Sur un utérus non cicatriciel, la fréquence de RU est estimée entre 1/17.000 et 1/20.000 accouchements [5]. Entre janvier 2012 et janvier 2017, un seul cas de rupture utérine spontanée sur utérus sain lors du déclenchement du travail par misoprostol a été enregistré dans notre service, soit une incidence de 1/13.537 accouchements. Cela se rapproche des chiffres décrits dans la littérature. Le misoprostol a été incriminé dans ce cas inédit dans notre service. Le misoprostol est un analogue synthétique de la prostaglandine E1. Initialement indiqué dans le traitement de l'ulcère gastroduodénal, il a rapidement vu ses champs d'utilisation s'élargir vers l'utilisation en obstétrique dans les avortements médicamenteux et le déclenchement du travail, malgré l'absence d'autorisation de mise sur le marché (AMM) dans ces indications. Le misoprostol a trouvé une place privilégiée en obstétrique dans les pays en voie de développement, étant donné son coût faible et sa facilité de conservation et d'administration, malgré ses nombreuses complications de type hypercinésie utérine, rupture utérine, ou arrêt cardiaque, retrouvées dans la littérature [6].

La particularité de notre cas clinique se trouve dans le terme précoce de la rupture (début du troisième trimestre), dans l'absence de facteurs favorisants ou prédictifs, dans la faible dose de misoprostol administrée (au total 100 μg). Les ruptures utérines spontanées rapportées surviennent essentiellement au cours du troisième trimestre. Elles peuvent cependant être observées plus précocement, comme dans notre observation [6]. Les facteurs prédictifs de rupture utérine sont nombreux, dont les plus importants sont l'utérus cicatriciel, l'utérus malformé, la multiparité, les manœuvres obstétricales, les extractions instrumentales, les dystocies mécaniques, les antécédents de curetage utérin, et l'utilisation d'ocytociques parmi lesquels figure le misoprostol [7]. Chez notre patiente, le déclenchement par misoprostol était le seul facteur de risque trouvé, ce qui a donné à cet accident un caractère totalement imprévisible. Le tableau clinique de la rupture utérine est généralement bruyant et les signes typiques sont les douleurs pelviennes violentes, une sensation de déchirement, les métrorragies, et l'instabilité de l'état hémodynamique évoluant vers l'état de choc [8]. Sur le plan clinique, notre patiente a présenté un tableau clinique tronqué fait seulement de douleurs abdominales avec des métrorragies minimes ce qui a induit à un doute diagnostique, la pauvreté des signes cliniques chez notre patiente sont expliqués par le fait que le ligament large est resté intact, jouant donc un rôle compressif, empêchant l'expansion de l'hématome et sa diffusion dans la cavité abdominale. Le tableau clinique étant trompeur, l'imagerie a joué un rôle important dans la démarche diagnostique.

La prise en charge thérapeutique des RU demeure une urgence médicochirurgicale et comprend une réanimation médicale qui sera suivie d'une exploration chirurgicale par une laparotomie. Le traitement chirurgical de la rupture utérine sur utérus sain doit être idéalement conservateur chez la femme jeune désireuse de grossesse, et consiste en une simple suture de la rupture. Dans le cas où le traitement conservateur paraît impossible à cause de l'étendue des lésions, une hystérectomie s'impose [1, 9]. Devant les ruptures négligées, le traitement conservateur est rarement possible. Dans notre cas, le choix de l'attitude thérapeutique était difficile, et le traitement conservateur a été décidé devant les constatations per opératoires et le désir de la patiente de garder une fertilité ultérieure. En cas de nouvelle grossesse, le risque d'une nouvelle rupture utérine varie de 4 à 19 % selon les séries [2]. Pour la plupart des auteurs, ce risque est plus élevé en cas de cicatrice corporéale qu'en cas de cicatrice segmentaire [10]. Il faudra dans ce cas assurer un suivi rapproché et prévoir un accouchement programmé par césarienne prophylactique à 38SA [9].

## Conclusion

Les prostaglandines, dont le misoprostol, doivent être utilisées avec prudence et avec une surveillance étroite, dans les IMG du 2^ème^ et 3^ème^ trimestre. Le tableau clinique est généralement bruyant, mais des formes incomplètes voire pauci symptomatiques peuvent se voir. Tout symptôme inhabituel devra faire penser au diagnostic et pousser à s'aider de l'imagerie. Des études ultérieures sont nécessaires afin de déterminer dans ces situations, le protocole idéal et la dose minimale efficace de misoprostol.

## Conflits d’intérêts

Les auteurs ne déclarent aucun conflit d’intérêts.
